# Risk prediction models for intensive care unit-acquired weakness in intensive care unit patients: A systematic review

**DOI:** 10.1371/journal.pone.0257768

**Published:** 2021-09-24

**Authors:** Wei Zhang, Yun Tang, Huan Liu, Li ping Yuan, Chu chu Wang, Shu fan Chen, Jin Huang, Xin yuan Xiao

**Affiliations:** 1 Department of Neurosurgery Intensive Care Unit, The First Affiliated Hospital of Wannan Medical College, Wuhu, China; 2 Department of Blood Purification Center, The First Affiliated Hospital of Wannan Medical College, Wuhu, China; 3 Department of Nursing, The First Affiliated Hospital of Wannan Medical College, Wuhu, China; Universidad Miguel Hernandez de Elche, SPAIN

## Abstract

**Background and objectives:**

Intensive care unit-acquired weakness (ICU-AW) commonly occurs among intensive care unit (ICU) patients and seriously affects the survival rate and long-term quality of life for patients. In this systematic review, we synthesized the findings of previous studies in order to analyze predictors of ICU-AW and evaluate the discrimination and validity of ICU-AW risk prediction models for ICU patients.

**Methods:**

We searched seven databases published in English and Chinese language to identify studies regarding ICU-AW risk prediction models. Two reviewers independently screened the literature, evaluated the quality of the included literature, extracted data, and performed a systematic review.

**Results:**

Ultimately, 11 studies were considered for this review. For the verification of prediction models, internal verification methods had been used in three studies, and a combination of internal and external verification had been used in one study. The value for the area under the ROC curve for eight models was 0.7–0.923. The predictor most commonly included in the models were age and the administration of corticosteroids. All the models have good applicability, but most of the models are biased due to the lack of blindness, lack of reporting, insufficient sample size, missing data, and lack of performance evaluation and calibration of the models.

**Conclusions:**

The efficacy of most models for the risk prediction of ICU-AW among high-risk groups is good, but there was a certain bias in the development and verification of the models. Thus, ICU medical staff should select existing models based on actual clinical conditions and verify them before applying them in clinical practice. In order to provide a reliable basis for the risk prediction of ICU-AW, it is necessary that large-sample, multi-center studies be conducted in the future, in which ICU-AW risk prediction models are verified.

## Introduction

Patients admitted to intensive care units (ICUs) are affected by both their illness and certain factors associated with their treatment, such as environmental changes, fear of death, passive compliance, and potentially permanent loss of function; consequently, a series of mental, physical, and psychological clinical syndromes may develop in such patients, such as depression, anxiety, delirium, and post-traumatic stress disorder [1]. Intensive care unit-acquired weakness (ICU-AW) is one such condition that may develop, which is generalized limb weakness caused by neuromuscular dysfunction that develops during critical illness. It involves a series of clinical symptoms, such as difficulty in weaning from mechanical ventilation, paresis or quadriplegia, decreased reflexes, and muscle atrophy [2]. It has been reported that the incidence of ICU-AW among ICU patients is 25% to 50%, and ICU-AW is associated with a prolonged duration of mechanical ventilation, a prolonged stay in an ICU, and increased mortality, all of which seriously affect the recovery of patients [3–5]. Currently, there are no specific methods and medicine for the treatment of ICU-AW, therefore, medical staff should pay attention to the prevention of ICU-AW. Early identification of ICU-AW risk groups and targeted intervention measures are of great significance for preventing the occurrence of ICU-AW [6].

Presently, ICU-AW is diagnosed by assessing the strength of a patient’s limb muscles. Generally, the Medical Research Council (MRC) scale is used to evaluate the strength of six pairs of bilateral muscles in a patient, including the muscles involved in wrist extension, forearm flexion, shoulder abduction, foot dorsiflexion, knee extension, and thigh bending [7]. However, due to the impaired consciousness or attention of a majority of critically ill patients who are admitted to an ICU, which may be caused by coma, sedation, or delirium, muscle-strength assessment is usually not possible in the early stages after a patient’s admission to an ICU. In addition, there is currently no consensus regarding a gold-standard method that can be used for the diagnosis of ICU-AW [8]. These factors that cause uncertainty with respect to ICU-AW identification and diagnosis may cause medical staff to ignore ICU-AW or delay the diagnosis of ICU-AW. Therefore, as soon as a patient is admitted to an ICU, in order to prevent a delay in ICU-AW diagnosis, it is important to quantify the risk of ICU-AW with the use of predictive models.

In previous studies, researchers have adopted different types of study design including single-center or multi-center for the development of ICU-AW risk prediction models. The risk prediction model of ICU-AW considers the multiple causes of ICU-AW, and through the use of statistical models, such a model may be employed to predict the probability of ICU-AW occurrence in ICU patients. On one hand, an ICU-AW prediction model can be used by medical staff to effectively screen high-risk patients for ICU-AW, improve their awareness of the risk of ICU-AW occurrence, and take corresponding preventive measures to reduce the risk of ICU-AW occurrence in ICU patients. On the other hand, it can also enable patients and their families to clearly understand the risk of ICU-AW occurring during a period of ICU admission and improve their understanding of and cooperation with work related to risk prediction and prevention of ICU-AW.

The purpose of this systematic review was to comprehensively search and review for studies regarding ICU-AW risk prediction models, in which such models had been developed and used to determine the risk of ICU-AW for ICU patients. It is worth noting that one previous study conducted a systematic review of 8 studies on risk prediction model of ICU-AW [9], and our study was a further exploration based on this systematic review, so there was the overlap in the two systematic reviews. However, the difference is that we have increased the amount of literature and analyzed the basic content of the model, method of development, the form of model, the applicability and limitations of the model in more detail, and provided an exhaustive summary of the characteristics, effectiveness and the differences between different ICU-AW risk prediction models that have been previously developed and applied. The findings of this study could consequently be used to make informed decisions regarding the use of one or more of these prediction models for the prediction of ICU-AW in ICU patients.

## Methods

### Inclusion and exclusion criteria

The inclusion criteria for this study were as follows: (1) cohort studies and case-control studies; (2) studies that regarded ICU patients older than 18 years old and in which patients had not been excluded on the basis of their race, nationality, or course of illness; (3) studies that involved the development of an ICU-AW risk prediction model for ICU patients and specific explanation of the tools used to diagnose ICU-AW and the main evaluation methods and steps; (4) studies that involved the internal and/or external verification of a prediction model after it had been developed.

The exclusion criteria were as follows: (1) studies that involved the analysis of the risk factors of ICU-AW for ICU patients but not the development of ICU-AW risk prediction models; (2) studies that involved the use of diagnostic tools whose reliability and validity had not been tested; (3) duplicate publications; (4) studies with incomplete data; and (5) non-Chinese- and English-language literature.

### Information sources and search strategy

This systematic review was carried out according to the Preferred Reporting Items for Systematic Review and Meta-analysis (PRISMA) statement and the Cochrane Handbook for Systematic Reviews (S1 Checklist) [10]. Prior to the execution of this systematic review, the Cochrane Library and other databases were searched to ensure that no similar systematic reviews had been previously published. The protocol was registered in PROSPERO (CRD42021244553). Available from: https://www.crd.york.ac.uk/prospero/display_record.php?RecordID=244553. Four English-language databases (PubMed, Embase, Cochrane Library, Scopus) and three Chinese-language databases (China National Knowledge Infrastructure, Weipu, and WanFang databases) were searched to collect studies regarding ICU-AW risk prediction models for ICU patients. The search considered all relevant studies that had been included in the databases from the time of inception of each database to March 2021. We used the search terms, which included terms in the Medical Subject Headings vocabulary and free terms for PubMed (S1 File) and the other databases. The search was restricted to English- or Chinese- language studies, and no limit was assigned with respect to the sample size. In addition, the reference lists of the included studies were searched to supplement the acquisition of relevant literature.

### Study selection and data extraction

All studies were first screened on the basis of their titles and abstracts. After obviously irrelevant literature had been excluded; further selection was carried out on the basis of the full text for a study to determine whether the study would eventually be included. After confirming the inclusion of the literature, we extracted the data by using the CHARMS checklist (the checklist for critical appraisal and data extraction for systematic reviews of prediction modeling studies) [11], which mainly includes the author(s) and publication year, country or region considered, study design, participants, method of development of a model, method of verification of a model, sample size, predicted outcomes, candidate factors, missing data, diagnostic tools for ICU-AW, incidence of ICU-AW, area under the receiver operating characteristic curve (AUROC), risk factors, and number and names of predictors. Two trained reviewers independently screened the literature and extracted the data, and disagreements were resolved by discussion and by reaching a consensus.

### Quality assessments

Two reviewers independently evaluated the quality of the included studies according to the Newcastle-Ottawa Scale (NOS) [12] and evaluated the risk of bias and applicability of the models using the Prediction model Risk of Bias Assessment Tool (PROBAST) [13]. In this method of evaluation of the risk of bias, if all fields for a model are rated as having a low risk of bias, the overall bias of the model is considered to be low; if more than one field is rated as having an unclear or high risk of bias, the overall risk of bias of a model is considered to be high. For the evaluation of the applicability of a model, if the study population, predictors, and results are consistent with the question considered in a systematic review, the applicability is considered to be high. If more than one area has low applicability, the overall applicability of the model is considered to be low.

### Statistical analyses

Descriptive analysis methods were used to summarize data regarding the general characteristics and method of development of the different prediction models and the predictive factors considered in these models.

## Results

### Description of search and eligible studies

**Fig 1:**
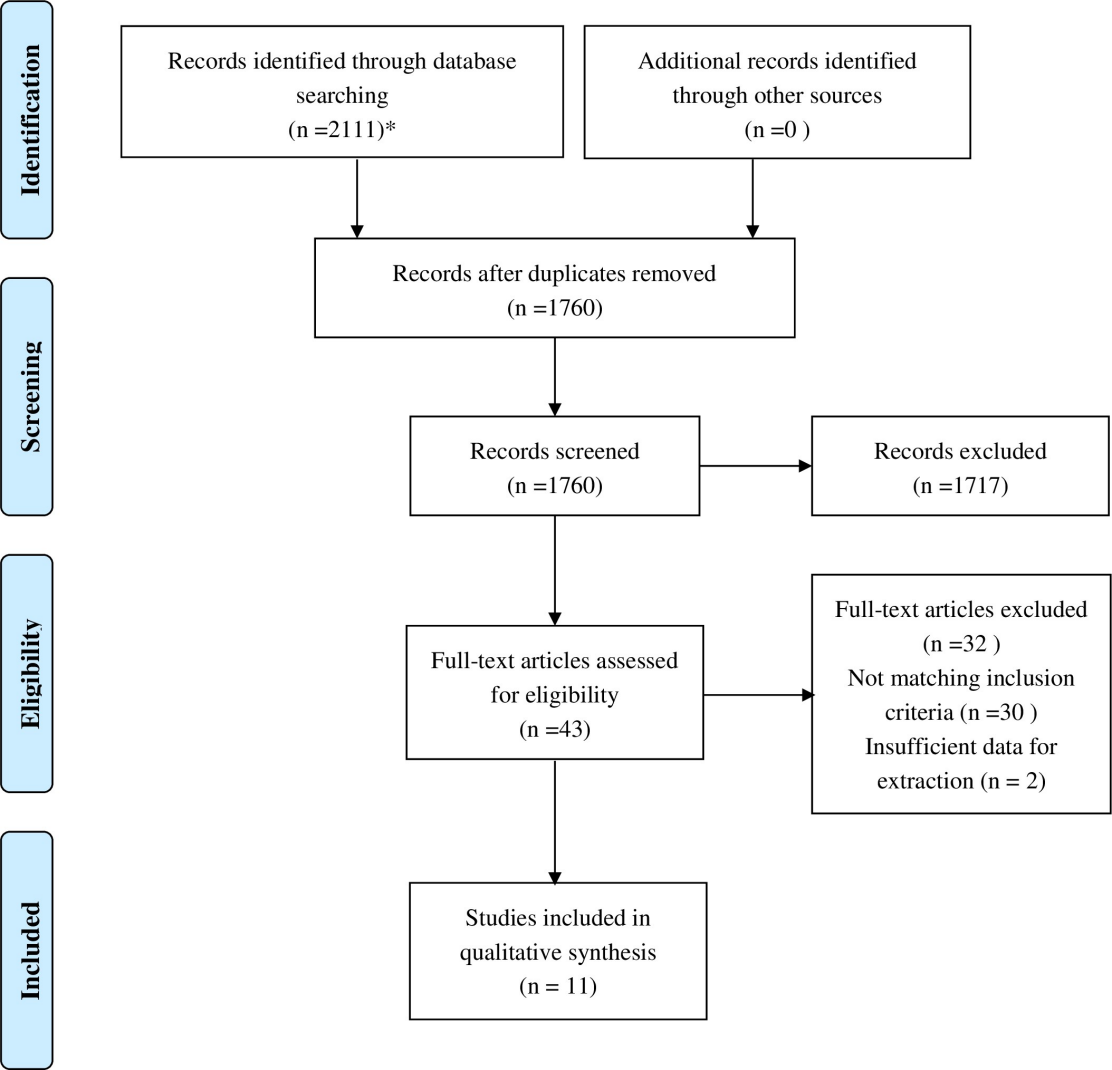
Flow chart of study selection.

A total of 2,111 studies were searched, and 1,760 studies remained after duplicates were removed. A total of 1,717 studies were excluded due to the obvious irrelevance of the topics of those studies, as determined by evaluating the titles and abstracts of the study papers. We identified 43 studies for further evaluation by reviewing the full-text of the articles; 30 studies were excluded because in those studies, the performance of prediction models had not been measured, and the method of development of the models was considered unreasonable; two studies were excluded because there was insufficient data in those studies for extraction. Finally, 11 studies that met the inclusion criteria for this systematic review were included. The detailed search steps have been described using the PRISMA 2009 Flow Diagram (Fig 1).

### Study characteristics and assessment of quality

A total of 11 studies involving 11 different ICU-AW risk prediction models with 5744 patients were included in this systematic review; of these, nine were English-language studies and two were Chinese-language studies. In terms of study design, five were cohort studies, two were observational studies, two were case-control studies, and two were retrospective studies. The NOS scores of the included literature ranged from 5 to 8 points, with indicates that the quality of the included literature was high. The basic characteristics and quality assessments of the included studies are presented in Table 1.

**Table 1:** Characteristics and quality of the included studies.

Study	Year	Country	Study design	Participant	Diagnosis tools	Predicted outcome	NOS
Ballve [14]	2017	Argentina	prospective cohort study	patients > 18 years and MV ≥ 24 hours	MRC	ICU-AW	6
Wolfe [15]	2018	American	retrospective study	patients > 18 years and 24 hours < MV < 72hours	MRC	ICU-AW	6
De Jonghe [4]	2002	France	prospective cohort study	MV ≥ 7 days	MRC	ICU-AP	7
Garnacho.Montero [16]	2001	Spain	prospective cohort study	patients with sepsis and MODS who aged 18 to 80 years and MV > 10 days	electrophysiologic testing	polyneuropathy	5
Hernández-Socorro [17]	2018	Spain	prospective observational study	patients required prolonged mechanical ventilation and stay at least 7 days in the ICU	QRF-US	neuromuscular acquired weakness	8
Pen˜uelas [18]	2016	Spain	retrospective study	MV ≥ 3 days and Glasgow Coma Score ≥ 10	definition based on expert consensus	ICU-AP	6
Witteveen [19]	2018	Netherlands	prospective cohort study	patients > 18 years; start MV at 48 hours after ICU admission	MRC	ICU-AW	7
Liu [20]	2017	China	case-control study	patients > 18 years; newly admitted ICU patients and MV ≥ 48 hours	MRC	ICU-AW	6
Wieske [5]	2014	Netherlands	prospective cohort study	newly admitted ICU patients and MV ≥ 2 days	MRC	ICU-AW	6
Weber-Carstens [21]	2009	Germany	prospective observational study	MV patients receiving analgesics and sedation; stay within 7 days after ICU admission	MRC	ICU-AP	5
Miao [22]	2021	China	case-control study	patients ≥18 years old; the hospital stay > 24 hours; the patient can move limbs according to instructions	MRC	ICU-AW	7

MODS: Multiple Organ Dysfunction Syndrome; MV: Mechanical Ventilation; MRC: Medical Research Council; ICU-AW: Intensive care unit-acquired weakness; ICU-AP: ICU-acquired paresis; QRF-US: Quadriceps Rectus Femoris Ultrasonography.

### Development of predictive models

The number of candidate predictors in each study ranged from 8 to 25, and in one study, continuous variables had been converted into dichotomous variables [14]. In the included studies, the total sample size ranged from 56 to 4157 cases, the number of result events ranged from 25 to 190, and the incidence of ICU-AW ranged from 2.7% to 68.5%. Four studies [4, 5, 16, 21] reported missing data, with the number of cases with missing data ranging from 8 to 12. For the development of the prediction models, logistic regression had been used in all studies. In five studies [4, 5, 14, 18, 21], backward stepwise selection had been used for the selection of predictor variables, and forward stepwise selection had been used in two studies [16, 19]. The details are listed in Table 2.

**Table 2:** Development of predictive models for ICU-AW.

Study	Candidate factors (n)	Sample size (n)	Result event (n)	Missing data (n)	Incidence of ICU-AW (%)	Method of developing the model	Variable selection
Ballve [14]	13	111	66	no data	40.5	logistic regression	backward stepwise selection
Wolfe [15]	8	172	80	no data	46.5	logistic regression	hierarchical entry of each variable
De Jonghe [4]	22	95	24	8	25.3	logistic regression	backward stepwise selection
Garnacho.Montero [16]	25	73	50	9	68.5	logistic regression	forward stepwise selection
Hernández-Socorro [17]	12	48	29	no data	60.4	logistic regression	select based on the algorithm of complete enumeration and Bayesian information criterion
Pen˜uelas [18]	13	4157	114	no data	2.7	logistic regression	backward stepwise selection
Witteveen [19^]^	23	349	190	no data	54.4	logistic regression	forward stepwise selection
Liu [20]	10	165	69	no data	41.8	logistic regression	select variables that are statistically significant of univariate analysis
Wieske [5]	20	212	103	7	48.5	logistic regression	backward stepwise selection
Weber-Carstens [21]	18	56	25	12	56.8	logistic regression	backward stepwise selection
Miao [22]	20	214*	39*	no data	18.2*	logistic regression	select variables that are statistically significant of univariate analysis
		92^#^	15^#^		16.3^#^		

*development of the model

#verification of the model.

### Performance and predictive factors of models

In eight studies [5, 14, 15, 17–20, 22], the discriminative performance of the prediction models was reported, with AUROC values ranging from 0.7 to 0.923, and the prediction performance reported in these studies was good. Seven studies reported the degree of calibration according to Hosmer-Lemeshow test [4, 5, 14, 15, 19, 20, 22], which the goodness of fit test of four models *P*> 0.05 [14, 15, 20, 22], and one of which was presented in the form of a calibration graph [19]. The classification ability of the models was reported in two studies, with a sensitivity of 74%–83.3% [16] and specificity of 88%–88.8% [21]. In terms of model verification, in three studies, internal verification was used to verify the established prediction models, and in one study, a combination of internal and external verification was used to evaluate the predictive effectiveness of the prediction model [19]. The final prediction models considered two to six predictors, and the predictor most commonly included in the models were age and the administration of corticosteroids. In five studies [5, 17, 19, 20, 22], formulas had been developed to determine scores for the risk of ICU-AW. The performance and predictors of all models are shown in Table 3.

**Table 3:** Performance and predictive factors of models for ICU-AW.

Study	Performance of models		Method of verifying the model	Predictors		The form of the model	Applicability and limitations
	AUROC Method of calibration			Number Name			
Ballve [14]	0.815	Hosmer-Lemeshow test	no reported	4	age, MV > 5 days, delirium, hyperglycemia > 3 days	MIP value is used as risk cutoff value, and MIP ≥ 36cmH_2_O associated with a high diagnostic to exclude ICU-AW	good applicability; a single center study and need to verify the results
Wolfe [15]	0.86	Hosmer-Lemeshow test	no reported	5	vasoactive medications, APACHE II score, hospital length of stay, age, early mobilization	No specific model proposed	good applicability; the results need to be evaluated in prospective study
De Jonghe [4]	no reported	Hosmer-Lemeshow test	no reported	4	female, the number of days with dysfunction of 2 or more organs, duration of mechanical ventilation, administration of corticosteroids	The OR value of each predictor is used as the risk estimate, which ranged from 1.10 to 14.90	good applicability; further research is warranted to determine the generalizability of results to all patients who undergo MV
Garnacho.Montero [16]	no reported	no reported	no reported	5	hyperosmolality, parenteral nutrition, use of neuromuscular blocking agents, neurologic failure, renal replacement therapy	No specific model proposed	general applicability; muscle biopsy was not systematically performed, so the contribution of myopathy cannot be evaluated
Hernández-Socorro [17]	0.902	no reported	no reported	2	QRF muscle area, QRF tendon thickness	formula for muscle wasting score based on the coefficient of each predictor	good applicability; unreported limitations
Pen˜uelas [18]	0.81	no reported	internal validation	6	steroid therapy, intensive insulin therapy, sepsis over the course of MV, acute renal failure, hematological failure, days of MV	coefficients were used to generate a weighted scoring system	good applicability; definition for ICU-AP was highly specific but low sensitivity; lack external validation
Witteveen [19]	0.7	Hosmer-Lemeshow test	external and internal verification	4	gender, RASS score, highest lactate, treatment with corticosteroids	formula for risk score based on the coefficient of each predictor	excellent applicability; the true incidence of ICU-AW in the validation cohort may be masked
Liu [20]	0.923	Hosmer-Lemeshow test	no reported	4	multiple organ failure, glucocorticoid, continuous renal replacement therapy, blood lactate level	formula for risk score based on the coefficient of each predictor	good applicability; lack multi-center external verification
Wieske [5]	0.71	Hosmer-Lemeshow test	internal validation	3	age, highest lactate levels, treatment with aminoglycoside in the first two days after admission	formula for risk score based on the coefficient of each predictor	good applicability; lack external validity verification
Weber-Carstens [21]	no reported	no reported	no reported	2	abnormal CMAP of peroneal nerve and pathologic spontaneous activity in tibial anterior muscle	abnormal dmCMAP was defined at a cutoff of < 3 mV as the classifying variable indicative for my opathy, which the predicted degree of paresis with an MRC of 3.8	general applicability; size of cohort is small, invasive operation is required and lack multi-center verification
Miao [22]	0.804	Hosmer-Lemeshow test	internal validation	4	age, gender, length of stay in ICU, sepsis	formula for risk score based on the coefficient of each predictor	general applicability; The sample size is small, and ICU-AW high-risk groups and low-risk groups are no detailed stratification analysis

MIP: Maximum Inspiratory Pressure; QRF: Quadriceps Rectus Femoris; dmCMAP: Compound Muscle Action Potential.

### Risk of bias and applicability evaluation of models

After the evaluation, we found that with respect to the domain of the participant, the risk of bias for all included studies was low, which the selected data sources were appropriate, and participants had been selected according to the inclusion and exclusion criteria. With respect to predictors, a high risk of bias was identified for two of the ten studies [4, 21], and the remaining studies had a low risk of bias. Ballve et al. [14], Wieske et al. [5] and Witteveen et al. [19] pointed out that the evaluator was blinded for the predictors, and the other of studies are "no information.” With respect to study results, we identified that two studies had a high risk of bias [4, 17], and nine studies had a low risk of bias. For a model-development study, if the number of events per variable (EPV) is < 10 there may be overfitting [23], and if the number of EPV is > 20, the study would be convincing [24, 25]. For model validation, if the number of EPV is < 100 [26], this may cause bias. Except for the sample size of the study by Witteveen et al. [19], the sample sizes of the other studies were insufficient and did not meet the requirements. In terms of variable selection, Liu et al. [20] and Miao et al. [22] directly performed multivariate analysis after univariate analysis without using appropriate variable-selection methods. In addition, the competition risk and time analysis of a prediction model had been considered in only one study [15], and the complexity of the data may have been overlooked in the other studies, in which no information had been provided in this regard. Except for the Witteveen et al. model [19], the other models had a high overall risk of bias. With respect to the applicability of the models, the applicability of all models was found to be good, and the study participants, predictors, and outcomes were highly consistent with those specified in the systematic-review questions. The evaluation of the risk of bias and applicability of the models, performed using PROBAST, is presented in Table 4.

**Table 4:** Risk of bias and applicability evaluation of models.

Study	Participant			Predictors				Outcome							Analysis										Overall risk of bias	Applicability
	①	②	risk of bias	③	④	⑤	risk of bias	⑥	⑦	⑧	⑨	⑩	⑪	risk of bias	⑫	⑬	⑭	⑮	⑯	⑰	⑱	⑲	⑳	risk of bias		
Ballve [14]	1	1	+	1	1	1	+	1	1	1	1	1	1	+	2	2	1	3	1	3	2	2	3	-	-	+
Wolfe [15]	2	1	+	1	3	1	+	1	1	1	1	3	1	+	2	1	1	1	1	1	2	2	3	-	-	+
De Jonghe [4]	1	1	+	1	3	3	-	1	1	1	1	2	3	-	2	1	1	1	1	3	2	2	3	-	-	+
Garnacho.Montero [16]	1	1	+	1	3	1	+	1	1	1	1	3	1	+	2	1	1	3	1	3	3	2	3	-	-	+
Hernández-Socorro [17]	1	1	+	1	3	1	+	1	2	1	1	3	3	-	2	1	1	1	1	3	3	2	1	-	-	+
Pen˜uelas [18]	2	1	+	1	3	1	+	1	1	1	1	2	1	+	2	1	1	3	1	3	3	1	1	-	-	+
Witteveen [19]	1	1	+	1	1	1	+	1	1	1	1	1	1	+	1	1	1	1	1	3	1	1	1	+	+	+
Liu [20]	1	1	+	1	3	1	+	1	1	1	1	3	1	+	2	1	1	1	2	3	2	2	1	-	-	+
Wieske [5]	1	1	+	1	1	1	+	1	1	1	1	1	1	+	2	1	1	2	1	3	2	1	1	-	-	+
Weber-Carstens [21]	1	1	+	1	3	3	-	1	1	1	1	1	3	+	2	1	1	2	1	3	3	2	1	-	-	+
Miao [22]	1	1	+	1	3	1	+	1	1	1	1	3	1	+	2	1	1	1	1	3	2	1	1	-	-	+

1: Yes / maybe; 2: No / probably not; 3: No information; "+": low risk of bias / high applicability; "−": high risk of bias / low applicability.

①Is the data source appropriate? ②Are the inclusion and exclusion criteria of the participant reasonable? ③Are the definitions and evaluations of predictors the same for all participants? ④Is the evaluation of the predictor performed without knowing the outcome data? ⑤Are predictors included in the predictive model valid? ⑥Is the classification of the results reasonable? ⑦Is the definition of the result reasonable? ⑧Does the definition of the outcome exclude predictors? ⑨Is the definition of results the same for all participants? ⑩Is the information about the predictor unclear when determining the result? ⑪Is the time interval between the predictor evaluation and the result determination reasonable? ⑫Is the sample size reasonable? ⑬Is the treatment of continuous and categorical independent variables appropriate? ⑭Are all included participants included in the statistical analysis? ⑮Are missing data included in the appropriate treatment? ⑯Does the use of single factor analysis to screen predictors avoided? ⑰Is the complexity of the data considered? ⑱Is the performance of the predictive model evaluated? ⑲Whether to consider the over-fitting, under-fitting and best-fitting of the predictive model? ⑳Are the predictors and their weights consistent with the reported results?

## Discussion

Due to the various adverse effects that ICU-AW may have on patients who are critically ill, ICU medical staff pay attention to the risk prediction of ICU-AW and interventions for its prevention and treatment. Recently, multiple studies regarding the development and verification of ICU-AW risk prediction models have been consecutively performed. However, there are differences between the final prediction models developed, verified, and used in the different studies; these differences may be caused due to the differences in the areas considered, participants included, and methods used in the different studies. Therefore, it is necessary to systematically evaluate the existing prediction models to provide a theoretical basis for medical staff to choose high-quality models that can be used as risk-screening tools for ICU-AW. Liu et al. [9] included eight studies about the risk prediction model of ICU-AW for systematic review, and the author believed that the current prediction model of ICU-AW had good predictive performance and applicability, but suggested that the whole process of model development and verification should be reported in a standardized way. The results of our study are similar to those of Liu et al. [9], but the difference is that we believe that although the prediction performance of most models is good, due to lack of reports, the prediction performance of some models is still uncertain. In addition, most of the model lack external verification, which the stability is unclear, and the further study is needed. However, based on the current results of this system review, which can provide readers with more information about the prediction model of ICU-AW, so that they can better choose a model suitable for the current clinical context.

Through this systematic review, we comprehensively searched for relevant studies regarding ICU-AW risk prediction models using evidence-based methods and conducted an objective quality evaluation. After screening, 11 studies were finally included, including nine model-development studies and one model-validation study. The AUROC values for seven models were ≥ 0.7, which shows that those seven models can effectively predict the risk of ICU-AW in ICU patients. In seven studies, the calibration performance of the developed prediction models had been assessed by using the Hosmer-Lemeshow test, which allowed for a more scientific development of the ICU-AW risk prediction models. However, most of the models were biased due to the lack of blindness, lack of reporting, insufficient sample size, ignoring of missing data, and lack of evaluation of the performance and calibration of the risk prediction models. Internal verification methods had been used in only three studies, and in one study, a combination of internal and external verification had been used to verify a prediction model. Therefore, it appears that research regarding ICU-AW risk prediction models is still in a developmental stage.

In terms of diagnostic tools for ICU-AW, in eight studies [4, 5, 14, 15, 19–22], the MRC scale was used to assess ICU-AW, and in the three remaining three studies [16–18], different methods to diagnose ICU-AW had been used, including electrophysiological testing, quadriceps rectus femoris (QRF) ultrasonography, and definition based on expert consensus. The use of the MRC scale for the diagnosis of ICU-AW was recommended by the official clinical-practice guidelines of the American Thoracic Association; if the MRC-scale score for an individual is lower than 48, a diagnosis of ICU-AW can be made [27]. However, it is usually difficult to control factors related to the evaluation of ICU-AW, including the assessor and assessment time, because there is a lack of professional rehabilitation specialists who can measure the muscle strength of the muscles of different muscle groups for patients. Furthermore, most patients who are critically ill are unable to cooperate with such evaluations due to severe disease, sedation, and delirium. In addition, electrophysiological examination cannot be used to distinguish whether the cause of muscle weakness is myogenic or neurogenic, and the relationship between muscle weakness and its causes is unclear. Moreover, the clinical practice applicability of an ultrasound scan in this context is unclear. However, the diagnostic tool of ICU-AW was only used as an evaluation content in this review, which was aimed to understand the current international trend of diagnosis of ICU-AW. Although there is uncertainty about the use of other diagnostic methods, the use of different diagnostic methods in different studies has no effect on the predictive performance of the model itself. Therefore, even if only using MRC score as a diagnostic criterion, and excluding studies that did not use MRC as a diagnostic methods, which the existing study results and conclusions also will not be changed.

Excluding duplicate predictors, in this systematic review, 28 predictors were considered; the predictors most commonly included in the studies reviewed were the administration of corticosteroids, age, highest lactate levels, and acute renal failure. The different studies considered different predictors because of the differences in the diseases types of the participants included, conceptual definitions, and diagnostic tools used in the studies. Corticosteroids, which are commonly used in ICUs, mainly refer to glucocorticoid drugs; an excessive intake of corticosteroids causes muscle dysfunction and nerve damage, promotes the decomposition of muscle-tissue protein, and leads to increased protein loss [20]. Moreover, the side-effect of corticosteroid use is lipodystrophy, and corticosteroids may increase both the uptake and turnover of fatty acids in adipose tissue, which are closely related to the occurrence of ICU-AW [28]. Age is an important risk factor for ICU-AW [29]. In elderly patients, muscle protein synthesis decreases and decomposition increases with age. A decrease in the amount of muscle proteins can directly cause muscle weakness, which may gradually develop into sarcopenia, thereby increasing the risk of ICU-AW [30]. Blood lactate level is another important predictor. On one hand, an increase in blood lactate levels reduces the pH of blood and makes it acidic, which can lead to the stimulation of muscle nerve endings and cause damage [20]. On the other hand, a high blood lactate level causes a decrease in ionized calcium (Ca^2+^) concentrations, which affects the release and reuptake of Ca^2+^ by the sarcoplasmic reticulum during the process of muscle excitation-contraction coupling [20]. This may cause a decrease in the excitability of muscle nerves and lead to the occurrence of ICU-AW. Previous studies have shown that increased levels of arterial blood lactate will cause a certain degree of damage to myocardial cells [31], and aggravating histiocellular ischemia and hypoxia [32], which will cause neurological disorders. In addition, the incidence of acute kidney injury (AKI) among ICU patients is high due to multiple risk factors such as sepsis, surgery, shock, diabetes, hypertension, heart failure, use of nephrotoxic drugs, etc., [33]. AKI can cause electrolyte disorders in patients, among which an elevated concentration of blood potassium is the most common. An elevated serum potassium concentration causes the depolarization of the cell membrane and leads to limb weakness and reflex disappearance. Thus it increases the likelihood of ICU-AW occurring in a patient.

The models considered in this study have certain advantages and disadvantages. The model by Witteveen et al. [19] has been externally verified and has good extrapolation. The models by Hernández-Socorro et al. [17] and Liu et al. [20] have better discrimination than the other models. However, some models have certain shortcomings. The predictors included in some models are not present when the patient is admitted to ICU, such as MV duration> 5 days and hyperglycemia > 3 days included in Ballve et al. [14]; hospital length of stay included in Wolfe [15]; duration of MV and days with organ dysfunction included in De Johnghe et al. [4], etc. Therefore these models can identify risk factors associated with the outcomes of ICU-AW, but may not be appropriate for early risk prediction of ICU-AW on ICU admission. In the study by De Jonghe et al. [4], only the degree of calibration of the model used was reported, and in another two studies [16, 21], only the specificity and sensitivity of the modes were reported. Hernández-Socorro et al. [17] used QRF ultrasonography to diagnose ICU-AW; however, it is not easy to measure the QRF muscle area and QRF tendon thickness, as compared to the ease of determining other predictor values. Garnacho-Montero et al. [16] and Weber-Carstens et al. [21] used electrophysiological testing to diagnose ICU-AW, but electrophysiological testing is susceptible to interference from factors such as a patient’s disease condition and electromagnetic interference caused by treatment equipment. When choosing an appropriate model for an actual clinical situation, medical staff should comprehensively consider the predictive performance of a model, the availability of predictive factors, and the convenience of outcome measurement.

### Limitations

This study has a few limitations. First, because this systematic review only included Chinese- and English-language literature, there may have been a publication bias. Second, in most of the studies included in this systematic review, only the development of the models was carried out and the studies lacked large-sample, multi-center external verification. Although most models have good predictive performances, the wide applicability and stability of the models need to be verified. Third, some models were developed for a long time, more than 10 years ago, and have not been calibrated and updated. Whether such models and the predictors considered in such models are suitable for current clinical-practice applications needs to be explored further. Finally, because of the heterogeneity of the data sources and methodology in the included literature, and because the method used for a meta-analysis of prediction-model studies has not been fully developed, the included literature has not been quantitatively analyzed.

## Conclusions

In this study, we considered a total of 11 ICU-AW risk prediction models, and systematically evaluated the model performance, methodological quality, method of development of a model, predictive factors, etc. The study results show that the predictive performance and applicability of most models are good, but the models lacked validity verification. This suggests that research regarding ICU-AW risk prediction models is still in a developmental stage, and there is no model that can be directly applied to the Chinese population. We suggest that prediction models with excellent performance and strong feasibility in all aspects should be developed in the future and their use for different populations from different regions should be verified.

## Supporting information

S1 Checklist PRISMA checklist. (DOC)

S1 File Search strategy. (DOCX)
