# Supplementary material for: Risk prediction models for intensive care unit-acquired weakness in intensive care unit patients: A systematic review
Source: PLoS One. 2021 Sep 24;16(9):e0257768. doi: 10.1371/journal.pone.0257768 (PMC8462700; doi:10.1371/journal.pone.0257768)
Supplement: S1 File — (DOCX) [file pone.0257768.s002.docx]

**Search strategy used for systematic review- PubMed**

((((("Critical Care"[Mesh] OR "Critical Care Nursing"[Mesh]) OR "Intensive Care

Units"[Mesh]) OR "Critical Illness"[Mesh])) OR ((((((critical care[Title/Abstract]) OR intensive care unit[Title/Abstract]) OR critical illness[Title/Abstract]) OR ICU[Title/Abstract]) OR intensive care[Title/Abstract]) OR critically ill[Title/Abstract])))

**AND**

(((("prognosis"[MeSH] OR "risk factors"[MeSH]) OR prediction[Title/Abstract]) OR predictor[Title/Abstract]) OR "risk"[MeSH] OR risk prediction[Title/Abstract])

**AND**

((((((((((((("paralysis"[MeSH]) OR ("polyneuropathies"[MeSH])) OR ("acquired polyneuropathies"[MeSH])) OR ("muscle weakness"[MeSH])) OR ("muscular diseases"[MeSH])) OR (paresis[Title/Abstract])) OR (weakness[Title/Abstract])) OR (myopathy[Title/Abstract] OR myopathies[Title/Abstract])) OR (neuromyopathy[Title/Abstract] OR neuromyopathies[Title/Abstract])) OR (polyneuropathy[Title/Abstract])) OR (neuromyopathy[Title/Abstract])) OR (Intensive Care Unit-Acquired Weakness[Title/Abstract] OR ICUAW[Title/Abstract])) OR (intensive care unit acquired paresis[Title/Abstract] OR ICUAP[Title/Abstract]))
